# Staphylococcal Scalded Skin Syndrome in a Ten-Month-Old Male

**DOI:** 10.7759/cureus.26975

**Published:** 2022-07-18

**Authors:** Margaux Baatz, Heather L Holley, John Ahlert, Maxwell J Rubin

**Affiliations:** 1 Osteopathic Medicine, Midwestern University Arizona College of Osteopathic Medicine, Glendale, USA; 2 Pediatrics, Midwestern University Arizona College of Osteopathic Medicine, Glendale, USA; 3 Pediatrics, Pleasant Pediatrics, Peoria, USA

**Keywords:** 10-month-old infant, staphyloccocus aureus, pediatric emergency, infectious disease, desquamation, staphylococcal scalded skin syndrome

## Abstract

A 10-month-old male presented with rhinorrhea and decreased oral intake and was diagnosed with an upper respiratory infection. Two days later, he returned to the clinic due to a lack of improvement and the onset of new symptoms, including facial edema and perioral skin irritation. That evening, he became febrile at 100.4 °F and went to the emergency department at the local children’s hospital. No further workup was done and the parents were instructed to continue with the current treatment regimen. Over the next 48 hours, the patient’s symptoms worsened with the new onset of bilateral extremity edema and desquamation. The patient was returned to the emergency department. A physical exam was notable for a blanching, desquamating, erythematous rash on the face and creases of the arms, legs, and groin. A positive Nikolsky sign was reported. A clinical diagnosis of staphylococcal scalded skin syndrome (SSSS) was made, and the patient was started on intravenous clindamycin. This case illustrates a severe presentation of SSSS in a pediatric patient, demonstrating the challenges it poses to diagnosis and treatment.

## Introduction

Staphylococcal scalded skin syndrome (SSSS), also known as Ritter disease, is a bacterial toxin-mediated skin disorder classically seen in children, most often those under the age of six, with a peak between ages two and three [1]. The average annual incidence of SSSS in the United States is approximated at eight cases per million children, with the majority of cases seen in children under the age of two [2]. SSSS occurs when specific *Staphylococcus aureus* exotoxins disseminate to the epidermis. *S. aureus* phage group 2 strains 55 and 71 are the most common subsets of the bacteria implicated in SSSS [1]. These strains have the ability to produce exfoliative toxin A (ETA), exfoliative toxin B (ETB), or both [1]. ETA and ETB are pathogenic toxins that disrupt keratinocyte attachments, resulting in progressive cutaneous erythema and desquamation [1]. Intrinsic serine protease enzymatic activity allows these toxins to cleave desmogleins, the desmosomal linking proteins responsible for adhesion between keratinocytes in the stratum granulosum [1]. Hematogenous dissemination of these exotoxins, from the initial source of *S. aureus* infection, elicits separation of epidermal keratinocytes and detachment of the superficial epidermis.

In children, SSSS typically arises from a focused site of infection such as otitis media, impetigo, bacterial conjunctivitis, or iatrogenic wounds; and, more often in neonates, the umbilical and diaper areas. The incubation period from the original *S. aureus* infection to SSSS ranges from 1 to 10 days [1]. Transmission of exotoxin-producing strains of *S. aureus* occurs among individuals, including those who are asymptomatic. Common locations of transmission include those of conglomeration, including newborn nurseries and neonatal intensive care units. It is suggested that the increased susceptibility in children is linked to the lack of protective antibodies against staphylococcal toxins, an increased amount of desmoglein-1 in the skin at a young age, or immature renal function preventing the excretion of exotoxins [2].

Although SSSS is seen more often in children, performing a PubMed literature search of case reports using keywords "staphylococcal scalded skin syndrome" and filtering to include infants aged birth to 23 months returned only 16 published case reports within the last ten years. Of the 16 case reports identified, several presented with a clear source of infection [3-8] or clear signs of infection upon initial presentation of a desquamating rash [9]. Here, we present a case of SSSS with an uncommon presentation as the only initial signs were cutaneous edema and erythema with a subacute onset of severe infection rather than the acute presentation that typically occurs with SSSS. The delayed onset of severe symptoms was likely, in part, the reason for the delayed diagnosis. A recent epidemiological study demonstrated that the prevalence of SSSS appears to be increasing; this case report aims to draw attention to the signs and symptoms of SSSS in hopes of timely recognition and treatment of SSSS in the future [2].

## Case presentation

**Figure 1:**
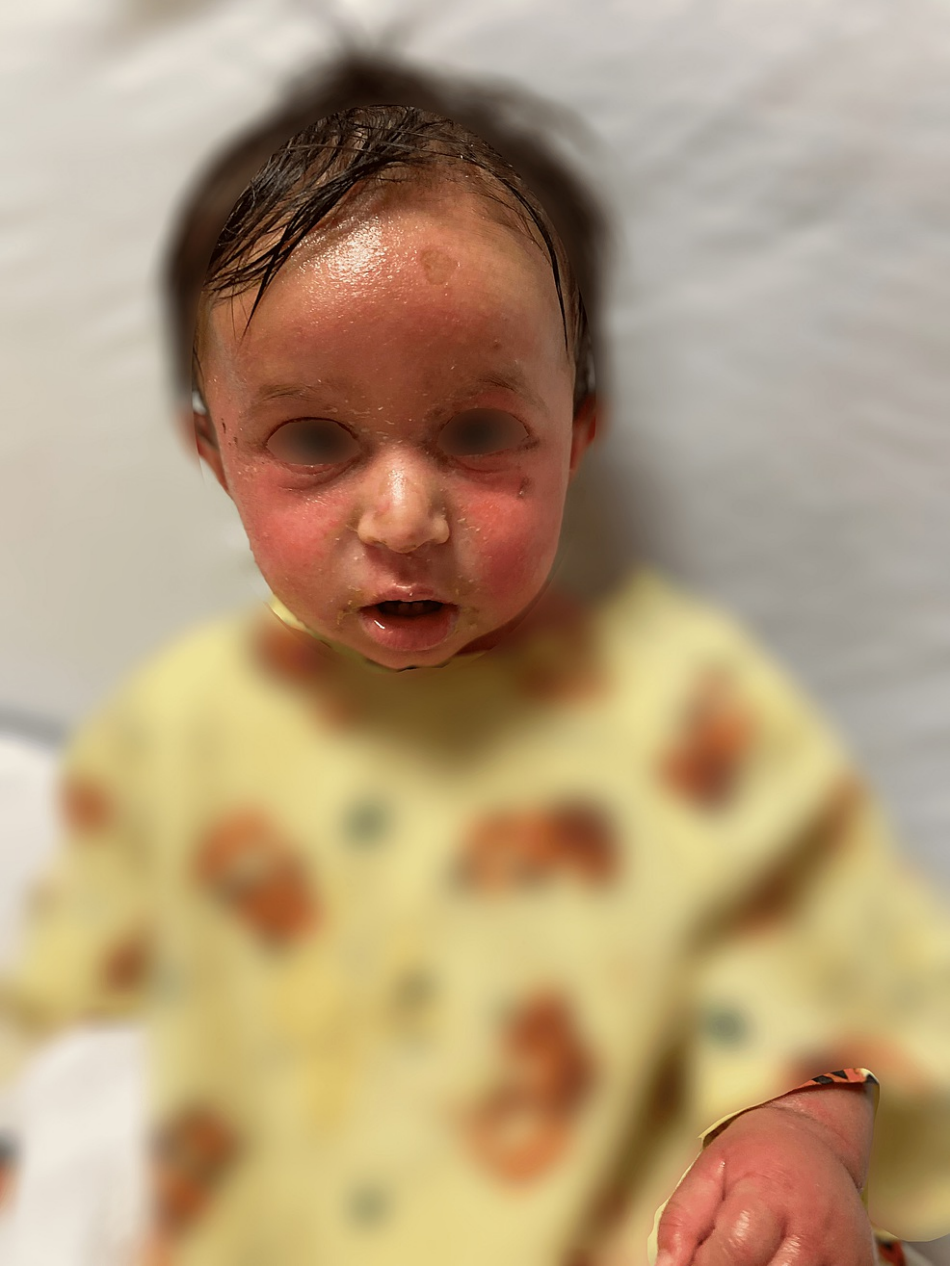
Patient on hospital day 1 shortly after initial onset of erythema, edema, and desquamation seen diffusely on the face as well as the right hand.

**Figure 2:**
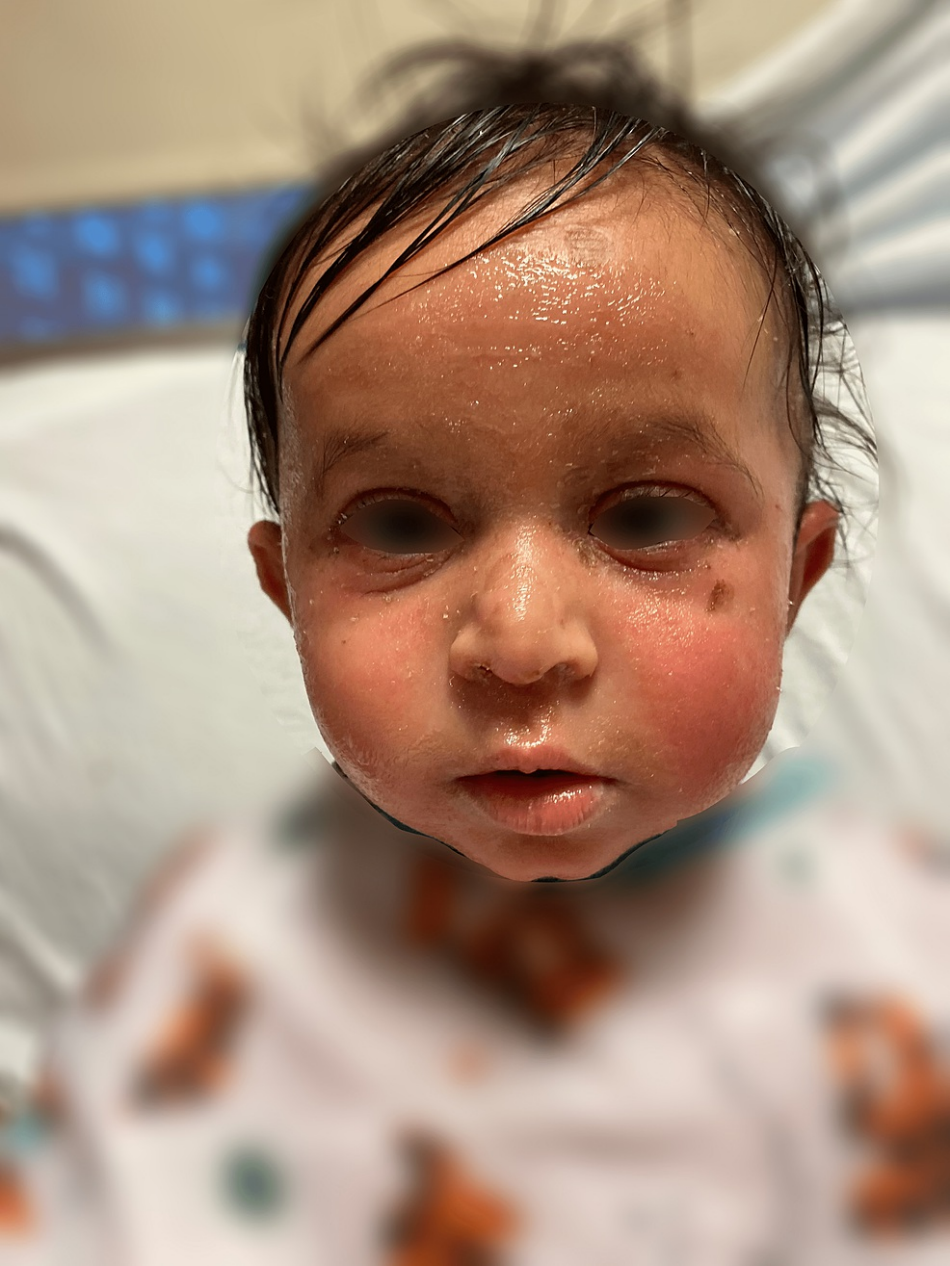
Patient on hospital day 2 after diagnosis with improved facial erythema, edema, and desquamation.

The patient was a 10-month-old male of Indian descent with a pertinent history of atopic dermatitis and egg allergy who presented to the outpatient clinic with rhinorrhea and decreased oral intake. No history of birth complications and immunizations were up to date. He was diagnosed with an upper respiratory infection at that time. Two days later, he returned to the clinic due to a lack of improvement and the onset of new symptoms, including diffuse facial edema and erythema, perioral skin irritation, and right-sided otalgia. Augmentin was given for the treatment of right-sided otitis media. Topical mupirocin was given for concern of impetigo, as the skin on his face was crusted on the right side and upper right perioral region. Rapid COVID-19 and Influenza A and B swabs were negative in the office. There was also a concern about an allergic reaction, and parents were informed to give an antihistamine as soon as possible. Additionally, they were instructed to monitor the patient’s facial edema closely and to return to the office the next day if symptoms had not improved. That evening, the patient’s parents reported that he became febrile at 100.4 °F and went to the emergency department at the local children's hospital. The patient's parents were instructed to continue with their current treatment regimen without further workup. Over the next 48 hours, the patient’s facial edema worsened with the new onset of bilateral extremity edema and diffuse desquamation. The patient was returned to the emergency department; initial vitals were notable for a heart rate of 137 and a blood pressure of 136/108. Laboratory results can be seen below in Table 1. A physical exam was remarkable for a blanching, peeling, erythematous rash on the face and creases of bilateral arms, legs, and groin (Figures 1-2).

**Table 1:** Laboratory values obtained upon arrival at the second visit to the emergency department. ESR: erythrocyte sedimentation rate, CBC: complete blood count, MCV: mean corpuscular volume, CMP: comprehensive metabolic panel, BUN: blood urea nitrogen, AST: aspartate transaminase, ALT: alanine aminotransferase.

Laboratory		Patient value	Reference range
Inflammatory markers			
	C-reactive protein	<0.4	<0.9 (mg/dL)
	ESR	9	<10 (mm/hr)
	Procalcitonin	0.08	<0.05 (ng/mL)
CBC			
	WBC	10.6	5.5–17.0 (k/mL)
	Hemoglobin	13.6	10.5–13.5 (g/dL)
	Hematocrit	41.5	33–39 (%)
	MCV	79	70–86 (fL)
	Platelets	551	140–450 (k/µL)
CMP			
	Glucose	87	55–110 (mg/dL)
	Na^+^	136	133–144 (mm/L)
	K^+^	4.9	3.6–5.2 (mm/L)
	BUN	< 5	5–25 (mg/dL)
	Creatinine	0.23	0.1–0.6 (mg/dL)
	Ca^2+^	10.9	8.8–11.2 (mg/dL)
	Cl^-^	104	98–108 (mm/L)
	HCO_3_^-^	23	17–29 (mm/L)
	Alkaline phosphatase (Alk P)	632	146–477 (U/L)
	AST	65	16–37 (U/L)
	ALT	30	30–65 (U/L)
	Total bilirubin	0.2	<0.8 (mg/dL)
	Total protein	6.5	3.6–7.4 (g/dL)
	Albumin	4.1	2.9–5.5 (g/dL)

**Figure 3:**
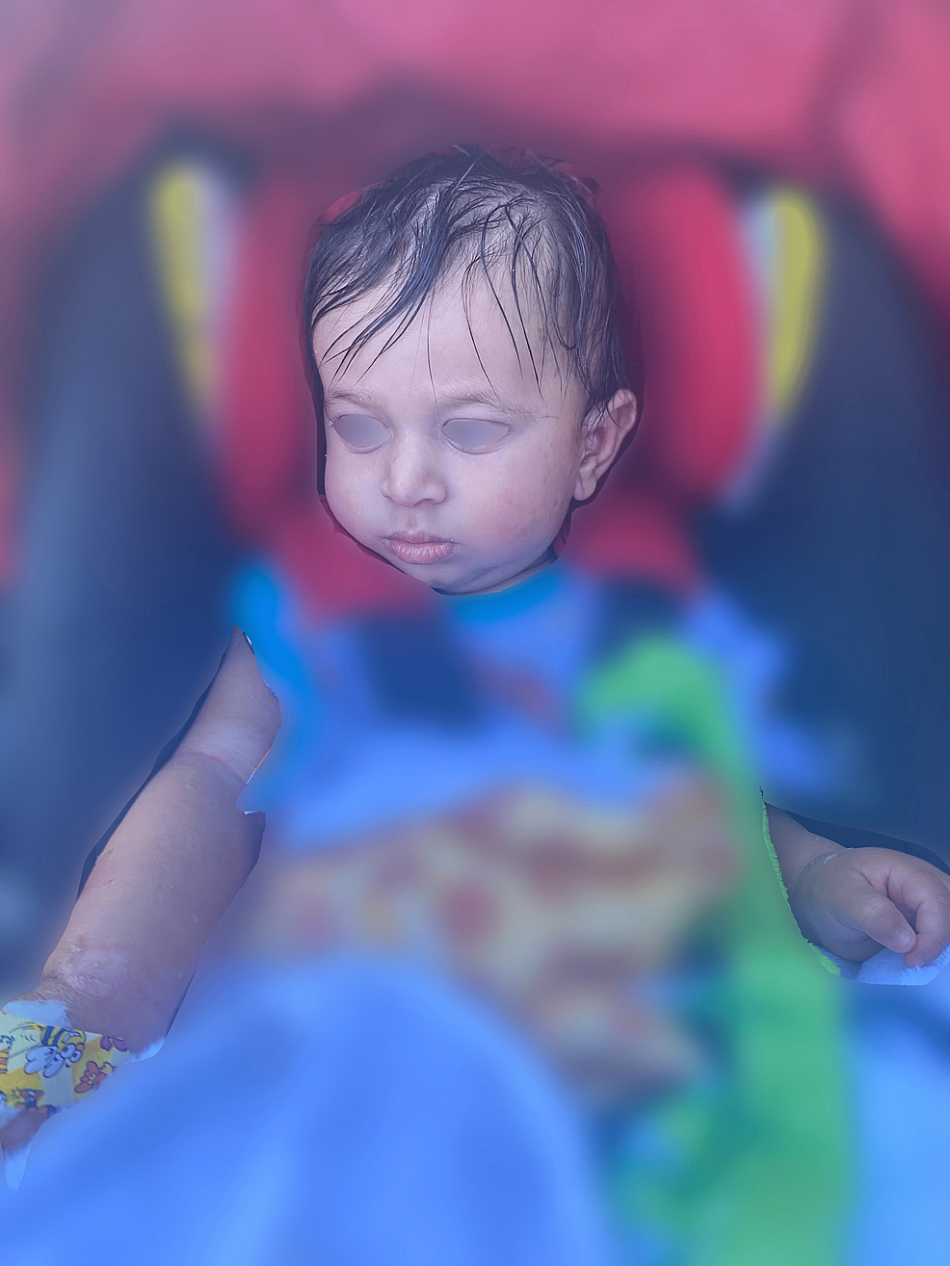
Residual skin desquamation seen on the right antecubital fossa and bilateral wrists.

A positive Nikolsky sign was reported. Of note, on physical examination, mucous membranes appeared moist without erythema or edema. A clinical diagnosis of SSSS was made and the patient was started on intravenous clindamycin. Bacterial cultures were later reported as negative. The patient was discharged two days later on oral Keflex with minimal residual desquamation (Figure 3).

Approximately a week after hospital discharge, the patient was seen as an outpatient and appeared to be recovering well without scarring or residual symptoms. Two months after the SSSS diagnosis, the patient tested positive for COVID-19 via rapid nasopharyngeal testing. Approximately two months after recovering from a COVID-19 infection, the patient underwent laboratory studies for possible immunodeficiency. Results are shown in Table 2. Since testing, the patient has been seen for a routine URI that resolved spontaneously with symptomatic treatment; there have otherwise been no further complications.

**Table 2:** Laboratory values obtained approximately four months after diagnosis of staphylococcal scalded skin syndrome.

Laboratory		Patient value	Reference range
Inflammatory markers			
	IgG	1079	553–1078 (mg/L)
	IgA	82	21–121 (mg/L)
	IgM	89	26–218 (mg/L)
	C-reactive protein	<3	<4.9 (mg/L)
	ESR	21	<10 (mm/hr)
	Lead	<2	<2 (mg/dL)
CBC			
	WBC	13	6–14 (k/mm^3^)
	RBC	4.7	3.80–5.40 (m/mm^3^)
	Hemoglobin	12	10.5–14 (g/dL)
	Hematocrit	37.7	32–42 (%)
	MCV	80.2	72–90 (fL)
	Platelets	389	130–450 (k/mm^3^)
	Segmented neutrophils	35.9%	-
	Lymphocytes	51.1%	-
	Monocytes	10.5%	-
	Eosinophils	1.8%	-
	Basophils	0.4%	-
	Immature granulocytes	0.3%	-

## Discussion

SSSS is often a clinical diagnosis based on history and physical examination. SSSS presents with progressive cutaneous erythema and desquamation associated with constitutional symptoms such as fever, irritability, malaise, and anorexia. Notably, on physical examination, mucous membranes are present normally and are typically moist, non-erythematous, and with patent nares. Sparing of mucosal membranes is often used as a distinguishing factor for SSSS. Other desquamating conditions such as Steven Johnson Syndrome (SJS) and Toxic Epidermal Necrolysis (TEN) can present with similar constitutional symptoms; however, their mucosal membrane involvement allows for differentiation from SSSS [1]. The lack of mucosal involvement seen in SSSS is due to the protein distribution throughout the skin. Desmoglein-1, specifically implicated in SSSS, is found in the upper epidermis but not within mucosal membranes [1]. On the other hand, desmoglein-3 is the desmosomal subtype most often found within mucosal membranes as well as the lower epidermis [1]. This property explains why mucous membrane involvement is not seen in SSSS.

Bacterial cultures that confirm the presence of *S. aureus* support the diagnosis of SSSS; however, these are not always attainable due to an unknown origin of infection or the presence of intact bullae that do not yield the causative organism. If cultures are obtained, they are usually negative in children, as in this case [1]. Many of the case reports reviewed reported negative or inconclusive cultures as well [3-7]. Literature suggests that routine cultures are not necessary unless the child is at risk for bacteremia, such as a febrile neonate, an immunocompromised child, or a child with a serious illness [1].

Management of SSSS consists of eradication of the causative staphylococcal infection and supportive care to promote healing, reduce discomfort, and minimize complications. The majority of patients are hospitalized for treatment, with typical interventions consisting of intravenous antibiotics and supportive care involving adequate hydration and wound care. Cephalosporins and anti-staphylococcal penicillins are the mainstays of treatment. Clindamycin, although not necessary for coverage, is commonly used in conjunction with these drugs because of its ability to inhibit *S. aureus* toxin production [5]. Flucloxacillin with clindamycin is the most commonly reported regimen in the literature [1,4-12].

A notable aspect of this case is the lack of immunodeficiency in this patient. Several case reports in the literature present cases of SSSS in infants who are days to weeks old, making it understandable why they may be immunodeficient as their immune systems have not yet had time to develop [5,7,8,10,13]. Additionally, aside from an elevated ESR, speculated to be in relation to the patient’s recent COVID-19 infection, his laboratory studies do not suggest immunodeficiency. We speculate that delayed diagnosis, likely due to the insidious onset of symptoms, as well as increased susceptibility, evidenced by his preexisting atopic conditions, contributed to the progression of this common skin infection to a desquamating dermatologic emergency.

## Conclusions

In the setting of pediatric dermatologic conditions, SSSS is a diagnosis that cannot be overlooked due to its associated morbidity and mortality risk. Patients should receive prompt antibiotics and supportive treatment to optimize patient outcomes. Presentation of symptoms includes those similar to other desquamating skin conditions such as SJS and TEN and is typically acute in onset. However, a high index of suspicion is essential to prevent progression, as in this case where symptoms were less acute in nature and lacked the characteristic skin desquamation on initial presentation. Case reports remain infrequent and no standard diagnostic or preventive strategy exists at this time. This case report brings attention to clinical features and a severe presentation of SSSS that can aid recognition in the future.

## References

[1] Leung AK, Barankin B, Leong KF (2018). Staphylococcal-scalded skin syndrome: evaluation, diagnosis, and management. World J Pediatr.

[2] Staiman A, Hsu DY, Silverberg JI (2018). Epidemiology of staphylococcal scalded skin syndrome in U.S. children. Br J Dermatol.

[3] Jindal S, Bhobhe M, Jerajani H (2012). An infant with skin rash. Indian Pediatr.

[4] Hennigan K, Riley C (2016). Staphylococcal scalded skin syndrome: a case review. Neonatal Netw.

[5] Mazori DR, Leonard A, Alexander JB, Glick SA (2020). The spectrum of staphylococcal scalded skin syndrome: a case series in children. Clin Exp Dermatol.

[6] Horna Strand A, Rubertsson S, Huss F, Mani M (2016). Epidermal exfoliation of over 95% after a burn in an 18-month-old boy: Case report and review of the literature. Burns.

[7] Gupta A, Jacobs N (2013). Visual diagnosis: 2-week-old has a red, peeling rash. Pediatr Rev.

[8] Franco L, Pereira P (2016). Staphylococcal scalded skin syndrome. Indian Pediatr.

[9] Campbell S, Crawford NW (2013). Varicella infection in infants less than 12 months. Vaccine.

[10] Singh SN, Tahazzul M, Singh A, Chandra S (2012). Varicella infection in a neonate with subsequent staphylococcal scalded skin syndrome and fatal shock. BMJ Case Rep.

[11] Conway DG, Lyon RF, Heiner JD (2013). A desquamating rash; staphylococcal scalded skin syndrome. Ann Emerg Med.

[12] Jordan KS (2019). Staphylococcal scalded skin syndrome: a pediatric dermatological emergency. Adv Emerg Nurs J.

[13] Bae SH, Lee JB, Kim SJ, Lee SC, Won YH, Yun SJ (2016). Case of bullous impetigo with enormous bulla developing into staphylococcal scalded skin syndrome. J Dermatol.

